# A novel insight into aging: are there pluripotent very small embryonic-like stem cells (VSELs) in adult tissues overtime depleted in an Igf-1-dependent manner?

**DOI:** 10.18632/aging.100231

**Published:** 2010-11-13

**Authors:** Mariusz Z. Ratajczak, Dong-Myung Shin, Janina Ratajczak, Magda Kucia, Andrzej Bartke

**Affiliations:** ^1^ Stem Cell Institute at James Graham Brown Cancer Center, University of Louisville, Louisville, KY 40202, USA; ^2^ Department of Physiology, Pomeranian Medical University, Szczecin, Poland; ^3^ Department of Physiology, Southern Illinois University School of Medicine, Springfield, IL, USA

**Keywords:** aging, longevity, IGF-1, RasGRF1, VSEL

## Abstract

Tissue and organ rejuvenation and senescence/aging are closely related to the function of stem cells. Recently, we demonstrated that a population of pluripotent Oct-4^+^ SSEA-1^+^Sca-1^+^Lin^-^CD45^-^ very small embryonic-like stem cells (VSELs) resides in the adult murine bone marrow (BM) and other murine tissues. We hypothesize that these pluripotent stem cells play an important role in tissue/organ rejuvenation, and have demonstrated that their proliferation and potentially premature depletion is negatively controlled by epigenetic changes of some imprinted genes that regulate insulin factor signaling (Igf2-H19 locus, Igf2R and RasGRF1). Since the attenuation of insulin/insulin growth factor (Ins/Igf) signaling positively correlates with longevity, we propose, based on our experimental data, that gradual decrease in the number of VSELs deposited in adult tissues, which occurs throughout life in an Ins/Igf signaling-dependent manner is an important mechanism of aging. In contrast, a decrease in Ins/Igf stimulation of VSELs that extends the half life of these cells in adult organs would have a beneficial effect on life span. Our preliminary data in long-living Igf-1-signaling-deficient mice show that these animals possess a 3-4 times higher number of VSELs deposited in adult BM, lending support to this novel hypothesis.

## INTRODUCTION

Senescence is an inevitable consequence of life. As a result of exposure to intrinsic- as well as extrinsic-aging factors, cellular aging is triggered by gradually accumu-lating DNA damage and epigenetic changes [1]. Thus, aging can be envisioned, at the cellular level, as a result of altered cell function in response to changes in DNA structure that directly affects proper gene expression. Since stem cells (SCs) have more efficient mechanisms of DNA repair, these changes affect mainly already differentiated somatic cells. SCs, however, also age, and according to the best evidence, there is a gradual age-related decrease in their self-renewal potential; at the molecular level there are concomitant changes, such as the well-known phenomenon of telomere shortening [2].

Several well-known risk factors, such as obesity, diabetes, and lack of physical activity that lead to atherosclerosis of the cardio-vascular system and impair the function of vital organs (e,g., heart, kidney, or brain), limit overall life span. On the other hand, it is obvious that all these risk factors somehow must ultimately have an impact on the basic units of tissue rejuvenation, which are SCs [2]. They can directly affect SCs or damage the niches in which these cells reside and thereby impair SC self renewal and differentiation. On the other hand, these risk factors may also lead to enhanced turn-over/proliferation of SCs, resulting in premature exhaustion of these cells.

Recently, our group demonstrated the presence of pluripotent Oct-4^+^ SSEA-1^+^Sca-1^+^Lin^-^CD45^-^ very small embryonic-like stem cells (VSELs) in adult murine tissues [3]. We envision that these pluripotent stem cells (PSCs) are deposited during early embryogenesis and reside in adult tissues as a backup for monopotent tissue-committed stem cells (TCSCs) that rejuvenate particular organs. Our data indicate that the pool of these cells decreases in adult murine tissues in an age-dependent manner [4]. We have also demonstrated that the proliferation and potentially premature depletion of these cells is negatively controlled by epigenetic changes of some imprinted genes that regulate signaling of insulin factors (Igf2-H19 locus, Igf2R and RasGRF1) [5]. Thus, these molecular data that link insulin/insulin growth factor-1 and -2 (Ins/Igf) signaling to the proliferation state of VSELs have implications for aging and senescence. It is well known, for example, that the level of insulin-like growth factor-1 (Igf-1) in blood plasma negatively correlates with longevity [6]. In this review, based on our preliminary data, we postulate a novel link between plasma Igf-1 level, aging, and the pool of VSELs residing in adult tissues.

We propose a novel hypothesis in which gradual decrease in the VSEL pool deposited in adult tissues due to chronic due to Ins/Igf signaling is an important mechanism of aging. We assume that an opposite beneficial effect on the size of the VSEL pool and life span would result from a decrease in stimulation by insulin factors. Our preliminary data in long- living Igf-1-signaling-deficient mice, which have a 3-4 times higher number of VSELs in bone marrow (BM), lends support to this novel hypothesis. Thus, our data somewhat reconcile aging and longevity by showing that i) signaling by Ins/Igf and excessive caloric uptake are related and ii) both of these factors have a negative effect on the number of pluripotent VSELs residing in adult tissues. Depletion of these PSCs in an Ins/Igf-dependent manner may lead to a premature decrease in VSELs and the regenerative potential of monopotent tissue-committed stem cells (TCSCs) that, as VSEL progeny, directly rejuvenate tissues.

### Heterogeneity of the stem cell compartment

Stem cells (SCs) possess the unique property of self renewal where, by symmetric division, they generate two daughter SCs and so maintain their number, and where, by asymmetric division, they give rise to one SC and one progenitor cell that will differentiate into somatic cells that are used up during life. However, clear evidence indicates that SCs are not all equal and form a hierarchy in their ability to specify into adult tissues [7]. While the most primitive SCs, the fertilized oocyte (zygote) or the first blastomers in the morula, are able to give rise to both embryo and placenta (totipotent SCs), cells isolated from the inner cell mass (ICM) of the blastocyst differentiate into embryonic tissues only (pluripotent SCs) [8].

PSCs from the ICM of the blastocyst form first the epiblast, which subsequently gives rise to all three germ layers [8]. Thus, the epiblast is the source of PSCs, multipotent SCs (MSCs) giving rise to tissues form one of the germ layers, and finally, most differentiated TCSCs (monopotent SCs). TCSCs are already restricted in their differentiation potential to cells for one tissue only (e.g., epidermis, intestinal epithelium, liver, skeletal muscles, or lympho-hematopoiesis) and in adult organisms are usually identified in several characteristic tissue locations (e.g., in the basal layer of epidermis and hair budge [epidermis], the bottom of intestinal crypts [intestine], around Herring ducts [oval stem cells in liver], around muscle fibers [satellite stem cells in muscles], and in endosteal areas [bone marrow hematopoietic stem cells]) [7].

Looking at the hierarchy of the stem cell compartment and its developmental specification, an important question emerges. Is the differentiation of SCs a “one way street”, where during embryogenesis all PSCs gradually disappear, giving rise to TCSCs or, alternatively, do some epiblast-derived PSCs survive beyond embryonic development into adulthood and remain as a “hibernating” backup population for TCSCs? This possibility would somewhat mimic the organization of the stem cell compartment in urodeles, where a population of the precursors of pluripotent blastema SCs is left dormant during development in adult tissues [9].

**Figure 1.**
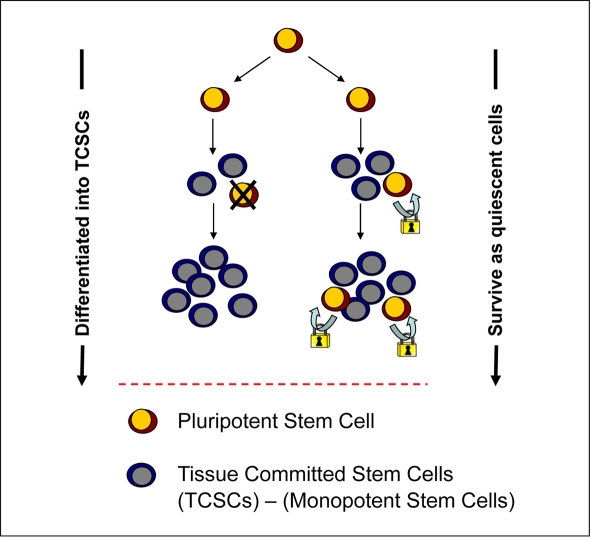
Potential VSEL contribution to tissue rejuvenation. VSELs deposited in adult tissues during embryogenesis/gastrulation may become eliminated after giving rise to TCSCs. Conversely, they may survive among TCSCs and serve as a potential backup/reserve source of TCSCs. Under homoestatic conditions, primitive VSELs should be protected from unleashing their own proliferation.

Identification of VSELs in adult tissues lends support to this second possibility, that some PSCs may survive into adulthood as a backup for rejuvenation of TCSCs (Figure 1). Thus, changes in the number of VSELs and their proliferative potential would directly impact aging/sense-cence and longevity by affecting the pool of TCSCs.

### Very small embryonic-like stem cells (VSELs) from adult tissues

We have purified VSELs by employing FACS-based multiparameter sorting, first from the murine BM and then for similar cells sorted from several other adult murine organs (i.e., brain, liver, skeletal muscles, heart, and kidney) [10]. Murine BM-derived VSELs: i) are very rare (~0.01% of nucleated BM cells); ii) are small in size (~3-5 μm); iii) express several PSC markers, such as Oct4, Nanog, Rex-1, and SSEA-1; iv) possess large nuclei containing unorganized chromatin (euchromatin); and v) are capable of differentiation into cells from all three germ lineages in vitro. Furthermore, VSELs exhibit a significantly higher nuclear/cytoplasm (N/C) ratio and a lower cytoplasmic area compared to HSCs. We recently confirmed the true expression of Oct4 and Nanog in BM-derived VSELs by demonstrating an unmethylated transcriptionally active open chromatin structure for both the Oct4 and Nanog promoters [5].

Corresponding populations of small (~4-7 μm) CD133^+^Lin^-^CD45^-^ cells that display embryonic-like morphology have been identified by us in human umbilical cord blood (UCB), mobilized peripheral blood (mPB), and adult BM [11]. Human VSELs, similarly to their murine counterparts, contain large nuclei that, by transmission electron microscopy, show the presence of primitive unorganized euchromatin and a relatively small rim of cytoplasm containing numerous round mitochondria. They also express Oct4 and Nanog within their nuclei and on their surface display the SSEA-4 antigen [11]. This suggests that a similar mechanism of tissue rejuvenation involving PSCs deposited in adult tissues also operates in humans.

We postulate that VSELs are a population of highly migratory cells. To support this claim, we observed that the number of VSELs increases, both in mice and in humans, during stress situations related to tissue organ injuries (e.g., heart infarct, stroke, or acute colitis) as well as after administration of certain drugs that are employed on a routine basis in the clinic to mobilize hematopoietic stem progenitor cells (HSCs) into PB (i.e., G-CSF and AMD3100) [12-14]. The high motility of these cells further supports the concept that they play an important role in tissue/organ regeneration.

### Evidence for VSEL pluripotency

Our data indicate that VSELs posses several morphological features of PSCs, such as a high Nuclear/Cytoplasmic ratio and the presence of unorganized euchromatin in nuclei. Below we will briefly discuss the most important other features of these cells that, based on their molecular analysis and in vitro and in vivo data, support their pluripotentiality.

#### Molecular evidence

PSCs, according to their definition, should, at the molecular level, i) express acknowledged markers of pluripotency (e.g., Oct4 and Nanog), ii) possess bivalent domains in promoters of develop-mentally crucial transcription factors (TFs), and iii) reactivate the X chromosome (in female PSCs), which becomes silenced at early stages of embryogenesis [15-16].

To support the notion that VSELs are pluripotent, our recent research has demonstrated that VSELs, not only express the transcription factor Oct4 (which is a characteristic marker of PSCs) at the mRNA and protein levels, but, as mentioned above, the Oct4 promoter in VSELs is in an active/open state [5]. Furthermore, our data indicate that murine VSELs display bivalent domains in promoters of several homeodomain-containing developmental TFs (ie.g., Sox21, Nkx2.2, Dlx1, Lbx14, Hlx9, and Pax5). The presence of transcriptionally active histone codes, such as H3K4me3, physically coexisting with repressive histone codes, such as H3K27me3, was confirmed by employing the carrier CHIP assay (submitted for publication). Finally, studies on female murine VSELs show that these cells partially re-activate the inactivated X chromosome. Thus, VSELs possess several molecular features expected from PSCs.

#### In vitro evidence

PSCs, according to their definition, should also differentiate into cells from all three germ layers (endo-, meso- and ectoderm) in vitro [7]. To support the concept of pluripotentiality of VSELs in vitro, we have also successfully developed several co-culture condi-tions where VSELs, if plated over supportive cell lines, may expand/differentiate into a variety of somatic cell types [17]. One of these is a co-culture system over OP9 stromal cells that allows hematopoietic specification of VSELs [18]. Another is a co-culture of VSELs over the myoblastic cell line C2C12, which allows some VSELs to differentiate and form spherical structures that resemble embryoid bodies and stain positively for the fetal isoform of alkaline phosphatase [19]. More importantly, cells isolated from these murine VSEL-derived spheres, if plated into cultures promoting tissue differentiation, expand into cells from all three germ-cell layers [3]. Interestingly, the formation of spheres by VSELs was associated with a young age in mice, and no sphere formation was observed with cells isolated from older mice (>2 years old). Unfortunately, we have so far not been able to reproduce this phenomenon using human VSELs, which suggests the involvement of murine cell-specific factors. In summary, while VSELs do not display all the molecular markers characteristic of PSCs, they are able to differentiate into cells from all three germ layers in vitro.

#### In vivo evidence

Our recent collaborative work in vivo indicates that VSELs may also be specified in vivo into MSCs [20] and cardiomyocytes [21-22]. Accordingly, in the first study by Taichman et al, VSELs isolated from GFP^+^ mice were implanted into SCID mice and 4 weeks later the formation of bone-like tissues was observed. To further validate that VSELs exhibit true MSC activity (bone formation), stromal cells were harvested from Col2.3ΔTK mice and implanted into SCID mice to generate thymidine kinase-sensitive ossicles. At 1.5 months, the ossicles were injected with 2000 GFP^+^VSELs. At harvest, there was observed to be a co-localization of GFP-expressing cells with an antibody to the osteoblast-specific marker Runx-2, the endothelial marker CD31, and the adipocyte marker PPARγ. Based upon the ability of uncultured VSELs to (i) differentiate in vivo into mutiple mesenchymal lineages and (ii) to generate osseous tissues at low density, Taichman et al. proposed that this population of cells fulfills many of the required characteristics of MSCs [20].

In another collaborative study, BM-derived VSELs freshly isolated from GFP^+^ mice were injected into the hearts of mice that had undergone ischemia/reper- fusion injury [21]. After 35 days of follow-up, VSEL-treated mice exhibited improved global and regional left ventricular (LV) systolic function (by echocardio-graphy) and attenuated myocyte hypertrophy (by histology and echocardiography) in surviving tissue when compared with vehicle- treated controls. Since VSELs isolated from GFP^+^ transgenic mice were employed for transplantation, we could track the fate of injected cells in the myocardium. Although VSEL transplantation resulted in isolated new myocytes and capillaries in the infarct region, their numbers were too small to account for all of the observed benefits [22]. Thus, it is likely that, in this particular tissue injury model, some paracrine effects by transplanted VSELs played an additionally important role and we are now analyzing the profile of growth factors and cytokines secreted by these cells.

However, unlike PSCs from established embryonic stem cell lines, VSELs neither complete blastocyst development after injection into the developing blastocyst nor form teratomas after injection into immunodeficient mice. The explanation for this is, as we have reported, epigenetic modification of crucial imprinted genes, from which some (i.e., Igf2 and RasGRF1) are important for senescence and longevity [5]. This will be discussed in more details in the following paragraph.

Epigenetic changes in imprinted genes related to insulin factor signalin keep VSELs quiescent in adult tissues. It is well known that changes in Ins/Igf signaling have important implications for aging. Accordingly, i) Igf-1 signaling negatively regulates lifespan in worms, flies, and mammals [23] and ii) Igf-1 and insulin level in blood is regulated positively by caloric uptake [24].

**Figure 2.**
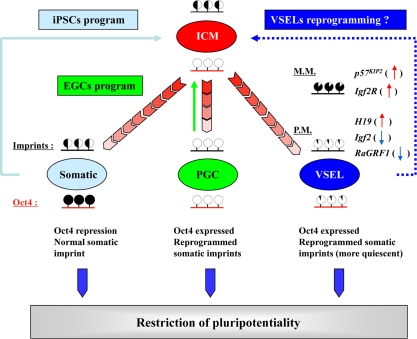
Reprogramming of genomic imprinting controls the pluripotentiality and quiescence of VSELs. During development, PGCs and VSELs undergo similar epigenetic reprogramming of genomic imprinted genes (black bar above the indicated cells); however, they also retain expression of some pluripotent genes (*e.g*., Oct4) through maintaining the corresponding promoter's DNA demethylation (red bar below the indicated cells). Intriguingly, VSELs undergo the parent-of-origin-specific reprogramming of somatic imprints, resulting in upregulated maternally expressed/proliferation-repressing imprinted genes (H19, p57^KIP2^, and Igf2R, up-red arrow) and down-regulated paternally expressed/proliferation-promoting genes (Igf2 and RasGRF1, blue-down arrow). In particular, the erasure of genomic imprints in these cells is responsible for preventing them from aberrant teratoma formation, but at the same time restrains their pluripotentiality. In contrast, differentiated somatic cells lose their pluripotency by turning off the transcription of pluripotent genes through stable DNA methylation of their promoters (*e.g.*, Oct4). However, they retain the somatic pattern of the genomic imprint. Thus, somatic cells may be dedifferentiated to PSCs by expression of pluripotent genes (blue box: iPSC protocol). In contrast, PGCs that express pluripotential genes, but erase the somatic imprint, may become pluripotent embryonic germ cells (EGCs) by proper remethylation of some of the erased imprinted genes (green box: EGC protocol). We hypothesize that similar modulation of parent-of-origin-specific reprogramming of somatic imprints in VSELs that enforces their quiescent state in tissues may “unleash” their pluripotentiality and reverse them to a fully pluripotent state (dark blue box: VSEL protocol). M.M.: maternally methylated loci; P.M.: paternally methylated loci.

What is crucial for our hypothesis and is the main topic of this review is that we have recently identified an important epigenetic mechanism that governs the VSEL quiescent state, preventing them from unleashing proliferation and the spontaneous growth of teratomas [5]. This mechanism is based on the epigenetic changes in selected somatic-imprinted genes (i.e., Igf2-H19, Ig2R, and RasGRF1) that are involved in insulin factor signaling (Igf-1, Igf-2, and Insulin) (Figure 2).

**Figure 3.**
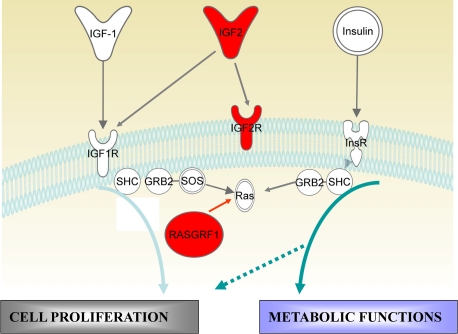
Insulin/Igf signaling and imprinted genes. In mammals there are three insulin factors (Insulin, Igf-1, and Igf2) that bind to two tyrosine kinase receptors, insulin receptor (InsR) and Igf-1 receptor (Igf-1R). Igf2R is a non-signaling mannose-type sink receptor for Igf2. Activation of InsR and Igf-1R lead, depending on cell type, to metabolic and proliferative responses. RasGRF1 is a small GTP exchange factor (GEF) that is involved in signaling from InsR and Igf-1R. VSELs show a decrease in Igf2 and RasGRF1 expression (blue) and overexpression of Igf2R (red) due to changes in the epigenetic state of imprinted genes. These epigenetic changes in genes regulating Insulin/Igf signaling keep VSELs quiescent in adult tissues. We hypothesize that chronic exposure to Igf/Insulin accelerates premature depletion of VSELs.

Accordingly, VSELs downregulate expression of Igf2 that, at the embryonic stage of development, is an autocrine growth factor for these cells, and in addition, upregulate expression of Igf2R, which serves as a mole- cular sink for Igf2 [25]. In contrast Igf2 may signal by binding to Igf-1R. Additionally, Ins/Igf signaling is impaired due to erasure of the imprint on RasGRF1, which is a small GTP exchange factor (GEF) for both Igf-1R and the insulin receptor (InsR) [26]. The involvement of these genes in signaling from activated insulin factor receptors is depicted schematically in Figure 3. Thus, as we believe, impaired signaling from Igf-1R and InsR is responsible for keeping these cells quiescent in adult tissues. We hypothesize also that chronic stimulation over time with insulin and Igf-1 could lead to accelerated depletion of these cells. Since the number of VSELs decreases with age in mice and correlates with their senescence, this could explain the aging process at the level of the PSCs that are deposited in adult tissues during development (VSELs) [4]. A decrease in these cells will impair the regenerative function of their progeny - the pool of TCSCs.

In fact, our studies performed on normal young (4-week-old) and old (2-year-old) mice revealed that the number of VSELs and their pluripotentiality decreases during ageing. Accordingly, VSELs from old mice show lower expression of the pluripotentiality master-regulators such as Oct4, Nanog, Sox2, Klf4, and cMyc and, at the molecular level, the Oct4 promoter in VSELs becomes hypermethylated with age and shows a closed chromatin structure. Furthermore, VSELs from old mice show the somatic type of methylation at both Igf2-H19 and RasGRF1 loci, which suggests that VSELs from old mice have increased sensitivity to insulin factor signaling. This suggests that chronic Ins/Igf signaling in VSELs may contribute to, age-related depletion of these cells.

**Figure 4.**
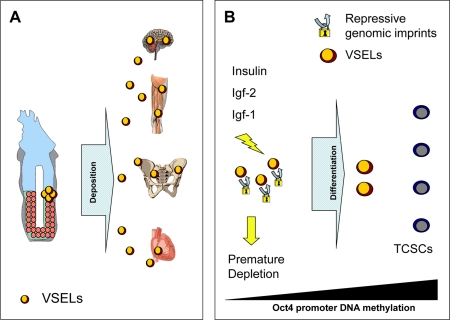
Hypothesis of developmental deposition of Oct4^+^ epiblast-derived VSELs in adult tissues and their depletion by chronic Insulin/Igf signaling. (**A**) Epiblast-derived VSELs are deposited in developing tissues as a backup population of SCs for production of TCSCs. (**B**) VSELs play a role in rejuvenation of tissues and organs as a source of TCSCs. Prolonged signaling by Insulin, Igf-1, and Igf-2 may lead to premature depletion of these cells (*e.g.*, due to high caloric uptake). By contrast, a decrease in Insulin/Igf signaling (*e.g.*, by caloric restriction) may ameliorate the age-related decrease in the number and pluripotency (*e.g.*, as indicated by the Oct4 promoter state) of these cells.

#### Lessons from Laron dwarf mice

To test the hypothesis that chronic Ins/Igf signaling may accelerate depletion of the pool of VSELs in adult tissues, we became interested in Laron dwarf mice. Due to a deficiency of growth hormone (GH) receptor, these animals display a severe reduction in the Igf-1 plasma level and do not display increase in GH-mediated Igf-1 plasma level in response to caloric uptake [27]. Interestingly, these animals live 30-40% longer than their normal littermates [28]. Several possible explanations for this effect have been proposed, but we became interested in whether a reduced level of Igf-1 in the plasma of these animals may directly impact the survival of VSELs in adult murine tissues.

Thus, to explain better the role of Igf-1 signaling in VSELs, we measured the number of VSELs in BM of murine Laron dwarfs by FACS. Interestingly, we noticed that the number of VSELs in the BM of plasma Igf-1-deficient Laron dwarfs is maintained at a 3-4-fold higher level than normal wild type littermates during aging. Our molecular analysis studies have additionally demonstrated that the Oct4 promoter in these animals shows also a higher level of demethylation [29].

Furthermore, while analysis of PB cell counts did not reveal any differences in the number of erythrocytes, platelets, and leucocytes between Laron dwarf mice and wild type controls, we observed that Laron dwarf mice have in BM i) a ~4-5-fold increase in the number of Sca-1^+^c-kit^+^lineage^-^ (SKL) hematopoietic stem cells and ii) a >4-fold higher number of clonogenic CFU-Mix, CFU-GM, BFU-E, and CFU-Meg cells.

Since the plasma Igf-1 level is regulated in mice by caloric uptake and in humans by calorie uptake if in particular protein content in the diet is low [30], these data shed new light on caloric restriction, senescence, and the size of hematopoietic stem cell compartment. Based on this, we propose a new paradigm in which chronic Igf-1 deficiency somehow protects VSELs from age-related elimination from BM. This mimics a situation seen in chronic caloric restriction in mice, where the Igf-1 level is low and results in longevity. Since the long-living Laron dwarf mice that maintain low levels of Igf-1 have higher numbers of VSELs and HSCs in BM, we postulate that chronically elevated levels of Igf-1, resulting, for example, from high caloric uptake, may lead to premature depletion of the stem cell pool, including VSELs and HSCs, and thus be responsible for premature aging in mice. This hypothesis is currently being tested in mice that overexpress GH and thus have high plasma Igf-1 level, and, interestingly, in contrast to Igf-1-deficient Laron dwarf mice, show a much shorter life span.

Further studies are also needed to link the effect of chronic high Igf-1 signaling in VSELs with the development of cancer. Of note, both murine Laron dwarfs [28] and human Laron dwarfs [31] with chronically low Igf-1 levels are significantly protected from developing cancer [28,31]. Since many human malignancies are activated by Igf -1 and Igf-2 signaling (e.g., due to loss of heterozygosity or loss of imprinting at the Igf-2-H19 locus) [32], we hypothesize that excessive activation of VSELs in an Ins/Igf-dependent manner could promote malignant transformation of these cells. However, the hypothetical reverse effect of low Ins/Igf signaling on a pool of VSELs that could become cancer initiating cells [33] requires further experimental study.

## CONCLUSIONS

Aging-associated changes in the nuclear architecture, chromatin structure, altered expression and activity of chromatin remodeling factors, and the change in pattern of epigenetic marks (DNA methylation and histone modification) affect all cells in the adult body, including the population of SCs that is responsible for proper tissue rejuvenation [34]. The elucidation of these precise mechanisms will help to develop more efficient anti-aging strategies as well as facilitate a better understanding of age-related risks for cancer genesis.

Based on our data, we postulate novel linkages between the Igf-1 level, aging, and the stem cell compartment. According to our hypothesis, early in development a population of VSELs would be deposited in developing organs as a backup for tissue-committed stem cells that plays a role in rejuvenation of tissues and organ regeneration after damage [7]. These cells seem to be protected from uncontrolled proliferation and age-related depletion by changes in imprinted genes that regulate insulin signaling [5]. We hypothesize that the pool of VSELs residing in adult tissues is regulated by the circulating Igf-1 level. An increase in Igf-1 level (e.g., resulting from a chronically high caloric uptake) would accelerate in an Ins/Igf -dependent manner an age-dependent depletion of the pool of VSELs and their potential to rejuvenate tissues. By contrast, a low Igf-1 level (e.g., as seen in Laron dwarf mutants or as a result of caloric restriction) would have an opposite and protective effect on these cells.

Thus, we present for the first time a hypothesis that reconciles aging, longevity, insulin factor signaling, and high caloric uptake with the abundance and function of pluripotent VSELs deposited in adult tissues. A decrease in the number of these cells will affect pools of TCSCs and have an impact on tissue rejuvenation and life span.
